# The impact of cultural beliefs and practices on parents’ experiences of bereavement following stillbirth: a qualitative study in Uganda and Kenya

**DOI:** 10.1186/s12884-021-03912-4

**Published:** 2021-06-25

**Authors:** Elizabeth Ayebare, Tina Lavender, Jonan Mweteise, Allen Nabisere, Anne Nendela, Raheli Mukhwana, Rebecca Wood, Sabina Wakasiaka, Grace Omoni, Birungi Susan Kagoda, Tracey A. Mills

**Affiliations:** 1 Department of Nursing, College of Health Sciences, Makerere University, P.O.BOX. 7072, Kampala, Uganda; 2 Centre for Childbirth, Women’s and Newborn Health, Department of International Public Health, Liverpool School of Tropical Medicine, Pembroke Place, Liverpool, L3 5QA United Kingdom; 3 Lugina Africa Midwives Research Network, Department of Nursing, College of Health, Makerere University, PO Box 7072, Kampala, Uganda; 4 Lugina Africa Midwives Research Network, C/o School of Nursing Sciences, University of Nairobi, PO Box 30197, Nairobi, Kenya; 5 Department of Geography and Environmental Science, Liverpool Hope University, Hope Park, Liverpool, L16 9JD UK; 6 School of Nursing Sciences, University of Nairobi, PO Box 30197, Nairobi, Kenya; 7 Mulago Specialised Women’s and Neonatal Hospital, P.O. Box 7051, Kampala, Uganda

**Keywords:** Culture, Qualitative, Stillbirth, Beliefs, Bereavement, East Africa, Experiences

## Abstract

**Background:**

Stillbirth is an extremely traumatic and distressing experience for parents, with profound and long-lasting negative impacts. Cultural beliefs and practices surrounding death vary considerably across different contexts and groups, and are a key influence on individual experiences, impacting grief, adjustment, and support needs. Few studies have explored cultural influences surrounding stillbirth in an African context. This study explored the influence of cultural beliefs and practices on the experiences of bereaved parents and health workers after stillbirth in urban and rural settings in Kenya and Uganda.

**Methods:**

A qualitative descriptive study design was employed. Face to face interviews were conducted with parents (*N* = 134) who experienced a stillbirth (≤ 1 year) and health workers (*N* = 61) at five facilities in Uganda and Kenya. Interviews were conducted in English or the participants’ local language, audio-recorded and transcribed verbatim. Analysis was conducted using descriptive thematic analysis.

**Results:**

Commonalities in cultural beliefs and practices existed across the two countries. Three main themes were identified: 1) *Gathering round, describes the collective support parents received from family and friends after stillbirth.* 2)*‘It is against our custom’* addresses cultural constraints and prohibitions impacting parents’ behaviour and coping in the immediate aftermath of the baby’s death. 3) ‘*Maybe it’s God’s plan or witchcraft’* summarises spiritual, supernatural, and social beliefs surrounding the causes of stillbirth.

**Conclusions:**

Kinship and social support helped parents to cope with the loss and grief. However, other practices and beliefs surrounding stillbirth were sometimes a source of stress, fear, stigma and anxiety especially to the women. Conforming to cultural practices meant that parents were prevented from: holding and seeing their baby, openly discussing the death, memory-making and attending the burial. The conflict between addressing their own needs and complying with community norms hindered parents’ grief and adjustment. There is an urgent need to develop culturally sensitive community programmes geared towards demystifying stillbirths and providing an avenue for parents to grieve in their own way.

## Background

A disproportionate burden of global stillbirth, defined as death of a baby before or during birth after 28 weeks’ gestation [1], affects low- and middle-income countries (LMICs). In sub-Saharan Africa and South Asia, including Uganda and Kenya, rates are around tenfold higher than high-income countries [2]. The death of a baby before or during childbirth is a traumatic and distressing experience for parents and families [3] associated with wide ranging and long-lasting negative health and social impacts. These include increased risk of recurrence, adverse mental health outcomes including anxiety, depression and post-traumatic stress disorder [4]. In LMICs, women may also experience chronic physical ill-health related to traumatic birth including obstetric fistula, in addition to the death of their baby. Culture, which is often defined as patterns of learned and shared behaviours and values evolving over generations within communities [5], is a key influence on acceptable conduct within societies. Culture influences ‘norms’ for spirituality, social and gender roles, attitudes towards family, work, health and illness [5, 6] and individual responses to adverse life events [5] including illness or bereavement [7]. Therefore, culture will affect parents’ reactions, decision making, coping mechanisms and the support available from others when a baby is stillborn [8]. Research surrounding the impact of stillbirth, mostly conducted in high-income countries (HICs), demonstrates that the death of a baby, before or during birth is a unique experience, associated with significant social stigma and isolation. This can invalidate parent’s grief experiences, hence prolonging and intensifying negative emotions. Lack of awareness and understanding amongst families and communities combine to reduce social support, which is recognised as a critical predictor of effective coping and adjustment [9].

Contrary to previous opinion, recent studies suggest that parents in sub-Saharan Africa experience similar psychological impacts and reactions to the death of a baby to those in other settings [3]. Less is known about key barriers and facilitators to parents’ coping and adjustment, but cultural beliefs and practices in these settings could amplify risks for negative experiences and outcomes after the death of a baby. In a study of community perceptions of stillbirth in rural Uganda, Kiguli et al., identified rapid burial with limited rituals and restrictions on public mourning for stillborn babies [10]. Mothers were often excluded from funerals and deprived of the collective support ordinarily expected after death of a loved one in African communities. The high value placed on fertility and expectation of motherhood as a predominant role for most women, may also be problematic for parents who experience perinatal death. In some communities, miscarriage (early pregnancy losses, often referring to fetal deaths before 28 weeks of gestation) [11] and stillbirth are widely believed to stem from bad omens, witchcraft or immorality and women who experience repeated losses are frequently reported to be shunned and even abandoned by their partners and families [12].

Understanding cultural and social influences on experiences in the immediate postnatal period after the death of a baby in LMICs is crucial to improving support for bereaved parents and challenging wider stigma surrounding stillbirth. There have been very few studies of parents’ experiences after the death of a baby in sub-Saharan Africa where the greatest numbers of perinatal deaths occur, particularly in urban communities, and health-workers’ perspectives are largely absent. This study aimed to describe the influence of cultural beliefs and practices on the experiences of bereaved parents and health workers after stillbirth in urban and rural settings in Kenya and Uganda.

## Methods

### Design, Participants and Study Setting

This study was conducted as part of a larger programme of work exploring experiences after stillbirth in sub-Saharan Africa and employed a qualitative descriptive design. Women and male partners who had experienced a stillbirth, in five urban, peri-urban and semi-rural facilities and surrounding communities in Kenya and Uganda within the previous 12 months, were recruited. In 2019, stillbirth rates for Kenya and Uganda were reported at 19.7 and 17.8 per 1000 births, respectively [13]. Most women give birth in health facilities, but postnatal care following discharge is extremely limited, partly due to focus on child health. Health workers including midwives, nurses and doctors who regularly provided care for bereaved women, in the same facilities were also recruited. In both countries, the research teams were supported by Community Engagement and Involvement (CEI) groups of parents who had previously experienced the death of a baby, and stakeholder groups including local academics, clinicians, and policy makers. CEI and stakeholder groups contributed to the research process at all stages from design to interpretation and dissemination [14].

### Participant selection and recruitment

Women were identified with the help of link midwives working at the facility maternity units. In Uganda, women who had birthed at home were additionally identified via village health team (VHT) members. Brief study information was provided and written consent to be contacted by the research team was sought. Not sooner than four weeks after the birth, research assistants contacted women to inquire whether they were interested in receiving more information. Those who agreed to participate were invited to an interview at a mutually convenient time, not sooner than six weeks after the birth. Male partners were recruited via women participants, midwives, VHTs and CEI group members. Where both partners in a couple agreed to participate in the research, interviews were conducted separately to provide a ‘safe’ space for free and open discussion around experiences, some of which might have related to the other partner. Health workers were informed about the research during meetings at the included facilities and information leaflets placed in the units. Those interested in participating, contacted the research team directly for more information. The sample size was guided by achieving data adequacy, in providing rich participant accounts across parents and health workers residing in urban and rural settings in the included countries. Data saturation was not prerequisite, although the sample size selected would be projected to be sufficient and no new codes were identified at the conclusion of analysis [15].

### Data collection tools and procedure

In-depth, individual, semi-structured interviews were conducted by trained research assistants at a place chosen by the participant; for women and partners this was mostly at home. Health workers were interviewed in a private room in their place of work. Topic guides were developed by the research team based on existing literature and reviewed by CEI groups in both countries, prior to use. Interviews with women and partners were conducted in the participants’ preferred language including English, Luganda, Kiswahili and Runyankole/Rukiga, health workers were interviewed in English. Interviews commenced with a broad opening question inviting participants to share experiences surrounding their baby’s death, for women: ‘Can you tell me about your pregnancy, the birth and the death of your baby’. Health workers were asked to describe their role and experiences with women and families after stillbirth. Additional focus was on exploring support and practices after the birth, the role of family, friends and communities including participant’s own beliefs and traditions and those they were aware of in their communities. A participant-led approach was adopted by allowing interrupted responses, and using prompts such as ‘can you tell me more about… or what makes you say… to clarify responses and explore in-depth where required. Field notes were made during and after each interview to capture nuances, including non-verbal cues. All interviews were audio-recorded, transcribed in the original language, and translated to English where necessary. Independent back-translation of a sample of translated interviews (around 10%) ensured accuracy of translation. Pseudonyms were used to protect participant identity. A study- specific distress policy was available to guide researchers in supporting participants discussing sensitive topics and emotions during data collection.

### Data Analysis

Due to the sensitive nature of the subject matter, transcripts were not returned to the participants for confirmability, however recapping was carried out at the end of each interview. Interview data were analysed thematically, using a four-stage approach, as described by Braun and Clarke (2006) [16]. Firstly, transcripts were read and re-read to ensure familiarisation with the data. Notes were made on the patterns and meanings in the data in relation to the research question. Formal manual coding was performed, and codes were organised into subthemes and main themes. Initial analyses were conducted by two researchers in each country and one UK based researcher and emergent themes discussed with the wider research team research to support rigour. Lastly, final themes were discussed, agreed upon and named as presented in the results with supporting quotes. The final interpretation was confirmed after presentation and discussion with the CEI and stakeholder groups in both countries.

## Results

### Demographic characteristics

A total of 195 interviews were conducted with 75 women (Kenya *n* = 44, Uganda *n* = 31); 59 male partners (Kenya *n* = 41, Uganda *n *= 18) and 61 health workers (Kenya *n* = 41, Uganda *n* = 20) from June 2017 to May 2019. Tables 1 and 2 present a summary of the demographic characteristics for the participants.

**Table 1:** Participant characteristics Parents (*N* = 134)

Women *n* = 75; Male Partners *n* = 59				
**Characteristic/Country**	**Kenya *n* = 85**		**Uganda *n* = 49**	
	Women *n* = 44	Partners *n* = 41	Women *n* = 31	Partners *n* = 18
**Age (years; median[range])**	28.5 [19–43]	31 [20–51]	25[19–40]	34[23–63]
**Years of education (median [range])**	12 [4–17]	15 [6–20]	10 [3–16]	11 [3–17]
**Urban dwelling n (%)**	19 (43%)	22 (54%)	18 (49%)	8 (44%)
**Married or cohabiting n (%)**	39 (89%)	40 (98%)	22 (72%)	16 (89%)
**Religion n (%)**				
Christian	44(100%)	38(93%)	23(74%)	7(39%)
Muslim		3(7%)	8(26%)	9(50%)
Other/None				(11%)
**No living children**	15 (34%)	15 (37%)	9 (29%)	0 (0%)
**Previous baby stillborn or neonatal death**	9 (20%)	5 (12%)	1 (3%)	2 (4%)

**Table 2:** Participant characteristics Health workers (*N* = 61)

Characteristic	*N* = 61			
Gender (n; %)	Female		Male	
	46 (75%)		15 (25%)	
Kenya	33 (80%)		8 (20%)	
Uganda	13 (65%)		7(35%)	
Role (n; %)	Nurse- Midwife	Midwife	Doctor	Other
	37 (61%)	12 (20%)	10 (16%)	2(3%)
Highest level of education (n; %)	Certificate	Degree	Postgrad degree	
	44 (72%)	13(21%)	4(7%)	
Time since qualification (years; median [range])	15(1–30)			

### Themes

Three main themes were identified from the interviews which described influences of cultural practices and beliefs on parent’s experiences after the death of a baby, the theme titles include direct quotes from participants. Collective support was illustrated in descriptions of families and friends ‘***gathering round’*** the bereaved parents; ***‘it is against our custom’*** described cultural constraints and prohibitions impacting parents’ behaviour and coping in the immediate aftermath of the baby’s death; spiritual, supernatural, and social beliefs surrounding the causes of stillbirth are summarised in **‘*maybe it’s God’s plan or witchcraft’***.

#### Gathering round

Families and friends played a central, and largely positive, role in providing emotional and practical support after the death of a baby by ‘gathering round’ mothers to care for them. Beyond offering condolences and comfort in the immediate period after the death, family members often took the lead in facilitating rituals, including arranging the funeral. This was often mandated by parents’ exclusion from involvement in burial of their own baby. However, some mothers, were physically unable to take part because of prolonged ill health resulting from pregnancy or birth complications. Family assistance ensured conventions could be followed and was highly valued by many parents:

*“My family buried my child; they obtained a birth certificate, and they were able to bury him. I still appreciate what they did because I was in no position to attend the burial.”* (**Bibi, mother, peri urban Kenya)**

Beyond the first days and weeks after the baby’s death, family and close friends continued to provide social and financial support to women, including help with domestic work, childcare, and provision of money to compensate for lost income. These networks were a crucial resource, allowing women and partners to focus on coping with the death of the baby and their own emotional recovery.

*“My friends took care of me, even my family members sent me some money and we managed to survive...They used to cook for me food, they could give me that warm massage, such things. They used to keep me company at home. They could come and sleep with me here to console me... It strengthens you in a way; you slowly start to forget what happened because whenever you are alone, you spend the entire time crying, but when you are with someone, she can bring up stories and you chat, and even laugh a bit”.* (**Nansubuga, mother, semi-rural Uganda)**

For male partners, the presence of extended family and female friends was additionally perceived as important in enabling them to return to employment and resume their primary role as provider for the family, as Maganda commented:

*“Her female friends and neighbours would visit her and comfort her. When we came back here, I only stayed with her for only two days and resumed duty at work. Her female friends and neighbours came and gave her company.”* (**Maganda, father, urban Uganda)**

Women living in urban settings in both Kenya and Uganda, particularly those who had migrated for employment or partners’ employment tended to describe feelings of loneliness and isolation more frequently after the death of their baby.

*“I expected my husband to tell his family, but he didn’t, and I have one brother who lives far so I too didn’t want to tell him. We even spoke this morning, but I didn’t want to tell him about it.”* **(Lilly, mother peri-urban Kenya)**

Barriers to accessing family included distance, transport costs and availability of mobile phones. In these circumstances some parents sought alternative ‘communities’ to avoid isolation, religion was an important focus and church members/ groups were very prominent in filling gaps.

#### It is against our customs

Cultural norms were cited as a key influence on the behaviour and actions of both parents and health workers after the death of a baby. These perceptions affected opportunities for seeing or having contact with the baby after the birth, whether the baby was named, mourning and burial rituals.

#### I didn’t see his face

For some parents, decisions around whether to see or have contact with the dead baby immediately after birth were influenced by fears of going against ‘customs’ and potential negative effects. Fears around future fertility or recurrent pregnancy loss were commonly cited as specific reasons for not having contact. Despite this, several women expressed regret for not having the opportunity to see or hold their baby, among few opportunities to create memories of their existence.

*“No, I didn’t. I didn’t get a chance to hold her. I think I was afraid, and I was not understanding anything, I was more confused and also it is against our customs to hold dead babies.”* **(Joy, mother, peri-urban Kenya)**

Assumptions of cultural prohibition meant that some health workers did not routinely offer the option of seeing or holding the stillborn babies to women. Others were conflicted in recognising the need to provide individualised care but had limited time and opportunity to support parents in making the ‘right’ decision that included being sensitive to cultural prohibitions. They expressed anxiety and uncertainty about whether their current practice was helpful:

*“...someone tells you; in our culture we are not supposed to look at the dead baby. You will not, when you are trying to [show them the baby] … much as you know the importance of them looking at the baby... you try to respect their culture and let them do what they feel is right.”* **(Lynette, health worker-Uganda)**

#### They don’t take it as a person

Participants in both Kenya and Uganda related community views and perceptions that babies who were stillborn did not have the same status as individuals who were born alive. Angella, whose baby was stillborn explained:

*“If it’s a stillbirth they don’t take it as a person, they do that only for a child who has at least cried after coming out of the womb. One who has cried is a human while the stillbirth isn’t regarded as a human since they haven’t heard its cry.”* **(Angella, mother, urban Uganda)**

This view of stillborn babies as ‘less human’ was further reinforced by use of the pronoun ‘it’ in some narratives and linked as a reason for the lack of normal rituals marking the baby’s existence. Although a few women, predominantly in urban Kenya, referred to their baby by name in the interview, naming a stillborn baby was relatively uncommon.

*“The baby has to first be born and he stays for about 2 days, that’s when they give him a name. One whom God takes away from earth after being born is the one who gets a name...But with the other one [referring to a stillborn baby] remember, he is born already dead, that one doesn’t get a name, he doesn’t.”*** (Obei, father semi-rural Uganda)**

This reluctance to assign a name was often rooted in the practice of passing down family or traditional names. Using familial names for a dead baby was considered to likely bring bad luck to the whole family, consequently naming ceremonies and baptism were not extended to stillborn babies even where parents were religious.

#### We do it differently

Parents and health workers in both countries described mourning rituals and funerals as being different for stillborn babies as compared to other deaths. Babies were buried very rapidly, often on the day after the birth, with only a few mourners present and a lack of normal traditions.

*“No condolence money is collected. No money is collected as condolence fee from the community members. After burial, no last funeral rites are done. No gathering and cooking is done after burial like for older people. They take it like as if you have not lost anyone.” ***(Mugisha, father, semi –rural Uganda).**

Participants associated rapid burial with secrecy and taboos surrounding stillbirth, one father in Kenya was told it was necessary to avoid the baby’s body being stolen and used for witchcraft. In some communities, fathers generally assumed responsibility for planning and conducting the burial:

*“We buried it [baby] without informing anyone else; me, my friend and my dad plus my brother who stays in Mbale. We proceeded and buried at that time without letting the neighbours know”. …where you bury, the grave isn’t supposed to be seen; you bury and just dig around it to camouflage around. You can even plant there something. Only family members are supposed to know and not all [family members] even because if you know Shafik is a witch doctor and has a bad heart; you don’t show[him].’’ ***(Shafik, father, urban Uganda)**

However, it was also commonplace for both parents to be excluded from funerals of stillborn babies. This was explained by concerns around future fertility or potential for recurrence of pregnancy loss. For example, women and partners were told that if a woman of reproductive age participates in the burial of a stillborn baby, she may never conceive again. Often, older relatives particularly women who were past childbearing age took the lead role.

*“For us, when a child dies, we [mother] aren’t supposed to bury them anyway…it’s a family affair.”* **(Hellen, mother, urban Kenya)**

*“In our culture, when a baby dies from the mother’s womb, we do it differently; it’s the elderly people that bury that baby, women who have reached menopause...”* **(Ali, father, semi-rural Uganda)**

Not being able to attend the funeral caused considerable distress for some parents. Amongst concerns was uncertainty as to whether the ‘necessary’ rituals had been performed and potential for future harm arising from omissions. For example, in central Uganda, traditions dictated that the umbilical cord should be cut off before burial of a stillborn baby and if this were not done, it was believed that the mother would not conceive again. Conversely, other women were forced by their circumstances to take a more active role in funerals than they wished. Lack of a willing partner, family support or resources to travel home meant women attending or conducting the burial personally. Failing to adhere to prescribed behaviour led to considerable anxiety around the potential impacts.

*“I felt bad about it in that up to now, it still worries me. Because as you know, culturally, people allege that I might not be able to give birth again...the fact that if the mother buries their baby, she will never conceive again in her life.”* **(Nalweyiso, mother, semi-rural Uganda)**

The place of burial, an important and sometimes contentious issue, depended on the culture and marital status of the parents. Patriarchal traditions across Kenya and Uganda dictated that children should normally be buried at their fathers’ ancestral burial grounds. These were sometimes located a distance away from the parents’ current residence and some lacked resources to transport the baby’s body. Access to family burial grounds was sometimes denied to single or separated women who also lacked financial resources to secure an appropriate burial and place to commemorate the baby’s life and existence.

*“...because they asked me where I was going to bury the child but I had nowhere, I didn’t have any single coin because even... So, there is a woman who had a banana plantation in the neighbourhood, and she said that a baby who hasn’t cried is buried in the middle of 2 banana plants. That was the only solution available at that time and I also didn’t have anything to do about it.” ***(Nalweyiso, mother, semi-rural Uganda)**

In urban Kenya, some facilities offered hospital-arranged funerals or access to public burial grounds for babies who were stillborn. These options were valued by parents facing financial pressures, geographical distance, and family disagreements over where to bury the baby, such as Jolly:

*“Yes, but you know I buried the baby not at our home or anywhere but at the cemetery, because there was a disagreement between our families. It was like the baby had died and among the [ tribe], they have their own cultures and we have our own cultures. I am a lady who was not married by that time and I could not take the baby to our home, and I could not take the baby to the father’s place, so we had to bury the baby at the [district]cemetery.” *(**Jolly, mother-peri urban Kenya)**

#### Maybe it’s God’s plan or witchcraft

Although participants acknowledged medical causes for stillbirth, spiritual, social and traditional beliefs were equally prominent. Religious beliefs featured conspicuously in most participant’s narratives and provided fundamental context for accounts of human experience. Stillbirth was frequently described in terms of ‘God’s plan’, with the lack of individual control over events offering comfort to women and partners, who often spoke of their ‘gratitude’ to God that the mother had survived.

*“We believe that whatever happens is a decision from God because we did all that we could; the scanning was done properly, we went through antenatal, took medications, she used to sleep under a mosquito net.” ***(Issac, father semi-rural Uganda) **

Religious perspectives were also often invoked by health workers when they offered support or reassurance to parents in the immediate aftermath of the baby’s death.

*“So [pause] I tell them that it’s not you who decided to get this pregnancy, it was God’s plan, he is the one who decided that you conceive and it’s the same God who has taken away this baby...”* (**Ritah, health worker, Uganda)**

However, not all participants took comfort from the notion that the baby’s death was predetermined by a higher power, several described their faith being tested. In these cases, religious fatalism contributed to feelings of powerlessness and lacking control over their lives which added to hopelessness and despair:

*“I thought about many things. I said, am I really the one who gives birth to children and they die? What did I do to you God?*” **(Evalyne, mother, urban Uganda) **

Participants in both countries also related widespread beliefs in their communities that supernatural forces were responsible for poor health outcomes including stillbirth. These beliefs unpinned links between natural disasters and poor pregnancy outcomes held in some communities:

*“Maybe even the earthquake which happened when I was pregnant. When one is pregnant and by any chance an earthquake occurs, you get a miscarriage and sometimes the baby dies in-utero. And when it occurs, many people lose their pregnancies.”* **(Mariam, mother, semi-rural Uganda)**

Stillbirths were frequently associated with witchcraft; this could occur directly as result of a woman’s involvement and her ‘possession’ by evil spirits, indirectly from ‘curses’ imposed by other ‘witches’, or failure to take herbal medicines. Having a stillbirth was often regarded as a bad omen, and some parents encountered suspicion and gossip in communities which added to stigma and isolation.

*“You know this is not a common thing, when it happens everyone is questioning in their mind what would have happened and they are not comfortable talking about it... and you believe that maybe your wife is a bad omen or you have been bewitched” ***(Ian, father peri urban Kenya)**

In both Kenya and Uganda, stillbirth was also considered to occur as a consequence of parent’s immoral behaviour. There were strong views that stillbirths were associated with infidelity or extra marital affairs:

*“This man went to see his wife and from there he went to see his ‘side chick’ [girlfriend] and she prepared tea for him and he took it to his pregnant wife in hospital and according to customs it is not right. And when you mix that way death must occur”. ***(Eric, father peri-urban Kenya)**

Several women and partners recalled hearing similar rumours circulating in their communities after the death of the baby which caused considerable distress. Some had heard of relationship breakdown and abandonment occurring as a result. Beliefs linking stillbirth with immorality were also shared by some health workers, potentially creating negative perceptions of parents experiencing the death of a baby:

*“… I understand that if you sleep with another man and you are pregnant then you will miscarry the baby or something bad will happen to the baby.” ***(Tamara, health worker, Kenya)**

## Discussion

This study has provided a uniquely in-depth account of the impact of cultural beliefs and practices on parent’s experiences in the initial days and weeks after the death of their baby before or during birth in Kenya and Uganda. We have confirmed the fundamental significance of cultural norms, intertwined with spiritual and social factors in influencing understanding, behaviour and support provided to parents after their baby died. Kinship ties, particularly relationships with the mother and father’s own parents, siblings, and in-laws (consanguineous and affinal kin) [17], provided the most powerful sources of support after the death of a baby. However, these crucial networks could be disrupted, for example by rural-to-urban migration, increasing isolation and loneliness for parents. The influence of wider communities was more mixed; norms surrounding the diminished status of stillborn babies and relative lack of rituals and mourning tended to undermine parents’ grief. Parents also described pressure to conform to traditional practices and the conflicts which arose if they chose to deviate from these for example to have contact with the dead baby. These tensions also impacted health workers’ practices in the period after the baby’s death. Although religious fatalism provided comfort for some parents, other common explanatory factors for stillbirth including supernatural forces, notably witchcraft and immoral behaviour, reinforced stigma and taboo.

The current study demonstrated that views and norms surrounding stillbirth in Kenya and Uganda were shaped by religion and traditional beliefs, alongside knowledge from modern medicine. These influences were often combined to underpin personal and wider community beliefs and practices. Falade (2019), highlights multiple and potentially competing sources of cultural authority in African societies, where high levels of individual commitment to Christianity or Islam co-exist with adherence to traditional rituals and are equally, if not more influential than science [18]. Close family relationships provided important and consistent sources of support to many parents, the primary reliance on relatives or close friends for emotional support after stillbirth is common across other low and high-income settings [19–22]. In Kenya and Uganda, material and financial support from families was often also required. Migration and urbanisation appeared to fragment family ties, and in these circumstances, some women were able to create new relationships to fill these gaps, these often involved or were facilitated through churches. The important role of religious groups in providing emotional support for individuals in time of health crises in Africa is frequently reported elsewhere [23].

Whilst consanguineal and affinal kin relationships were largely perceived as supportive, wider community influences, perceptions and practices around stillbirth could be more problematic. Stillborn babies were assigned lesser status, rarely named, and rapidly buried often without public mourning or parents’ presence. Similar practices have been reported in other LMICs settings including India [24] and had negative impacts, causing distress for parents who felt unable to express their grief openly. Kiguli et al. (2015) similarly described bereaved women in rural Eastern Uganda as ‘weeping in silence’ to maintain secrecy around the death of their baby [10]. Taboos around having contact with dead babies, deeply rooted in traditional beliefs in negative impacts on fertility and future successful pregnancy outcome prevented parents asking to see their baby, several later expressed unhappiness that this had not happened. Our findings demonstrate that health workers’ reluctance to challenge these conventions prevented offering choices to women and families, who were denied fleeting opportunities to create memories. Although some controversy exists over benefits, studies in HIC demonstrate that in the context of compassionate care, seeing and holding stillborn babies was associated with less subsequent psychological distress and that few women who chose to see their baby regretted doing so [25].

In addition to invalidating experiences, common explanatory beliefs often reinforced stigma, which occurs when individuals are labelled and stereotyped leading to isolation, status loss and discrimination [26]. Stillbirth was often seen as evidence of poor behaviour, particularly immorality or malevolent forces. Mothers, fathers and, sometimes, the wider family experienced public stigma as a result of stillbirth and particularly after multiple baby deaths. Self-stigma was also evident, where the fear of adverse community reactions after the death of the baby led women, in particular, to develop negative feelings which could decrease self-esteem and enhance social isolation [27]. Motherhood is identified as key expectation and central role for women in African societies and perinatal death, particularly where repeated, has been a trigger for marginalisation, relationship breakdown and sporadically, domestic abuse [28]. Similar to Haws et al.’s (2010) study in rural Tanzania, fatalistic beliefs in these communities relieved bereaved parents of some responsibility for the death of the baby, but only if they conformed to other conventions and expectations around conduct and behaviour [29].

## Strengths and limitations

Despite the importance of cultural beliefs in influencing coping and adjustment after stillbirth and huge potential psychological and social costs, few studies have addressed this issue in sub-Saharan Africa. This qualitative study explored cultural influences in a wide sample of parents, including women who birthed outside facilities and health workers in diverse urban and rural settings in Uganda and Kenya. This allowed exploration of perspectives across several tribal groups and major religious beliefs, including Christians and Muslims. However, considerable heterogeneity of norms, values and traditions affecting health behaviours have been identified within and beyond these two countries in sub-Saharan Africa [30], therefore our findings may not represent all East ‘African’ or ‘African’ experiences. Increasing facility-based birth means that health workers have assumed a crucial role in supporting women and families in the immediate period after stillbirth, therefore inclusion of their experiences here is a strength. Wider perspectives including those of community and religious leaders could enrich understanding further, nevertheless, feedback from National Institute for Health Research (NIHR) Stillbirth community engagement and stakeholder groups [14] in Malawi, Tanzania, Zambia and Zimbabwe demonstrated strong resonance with themes identified here, suggesting transferability to other African settings.

## Conclusion

Culture has a complex and powerful influence on parents’ experiences after the death of their baby in Kenya and Uganda. Aspects of African collectivist culture, such as close relationships amongst families were beneficial in enhancing support to enable parents to adjust after the trauma of stillbirth. However other norms and practices had more harmful impacts perpetuating stigma around stillbirth which is widely recognised to increase negative psychological and social outcomes for affected individuals. Stigma and fatalism also prevent open discussion of factors influencing perinatal death in communities, which has led to decades of neglect in driving improvement in stillbirth rates and care for parents in LMICs. Raising awareness of health consequences and challenging aspects of culture which have deleterious effects will be important to improve support for parents. This is a very sensitive issue, therefore interventions which promote collaboration and building trust with key stakeholders in communities are likely to be the most helpful. For example, community level education, engagement with local policy makers and religious leaders has been beneficial in challenging stigma and increasing uptake of programmes to prevent mother to child transmission of HIV in Sub Saharan Africa [31]. More sustainable progress may be achieved through strategies which accommodate positive health behaviours in the cultural framework, as opposed to direct criticism or dismissal [18].

## Data Availability

The data sets used and/or analysed during the current study are not publicly archived but are available from the corresponding author on reasonable request.

## Abbreviations

CEI: Community Engagement and Involvement
HIC: High- Income Country
LMIC: Low- and Middle-Income Country
NIHR: National Institute for Health Research
VHT: Village Health Team
